# Viral influencers: deciphering the role of endogenous retroviral LTR12 repeats in cellular gene expression

**DOI:** 10.1128/jvi.01351-24

**Published:** 2025-01-31

**Authors:** Veronika Krchlikova, Yueshuang Lu, Daniel Sauter

**Affiliations:** 1 Institute for Medical Virology and Epidemiology of Viral Diseases, University Hospital Tübingen 493697 , Tübingen, Germany; New York University Department of Microbiology, New York, New York, USA

**Keywords:** endogenous retroviruses, ERV9, LTR12, innate immunity, cancer, HDAC inhibitor

## Abstract

The human genome is like a museum of ancient retroviral infections. It contains a large number of endogenous retroviruses (ERVs) that bear witness to past integration events. About 5,000 of them are so-called long terminal repeat 12 (LTR12) elements. Compared with 20,000 human genes, this is a remarkable number. Although LTR12 elements can act as promoters or enhancers of cellular genes, the function of most of these retroviral elements has remained unclear. In our mini-review, we show that different LTR12 elements share many similarities, including common transcription factor binding sites. Furthermore, we summarize novel insights into the epigenetic mechanisms governing their silencing and activation. Specific examples of genes and pathways that are regulated by LTR12 loci are used to illustrate the regulatory network built by these repetitive elements. A particular focus is on their role in the regulation of antiviral immune responses, tumor cell proliferation, and senescence. Finally, we describe how a targeted activation of this fascinating ERV family could be used for diagnostic or therapeutic purposes.

## INTRODUCTION

**Fig 1:**
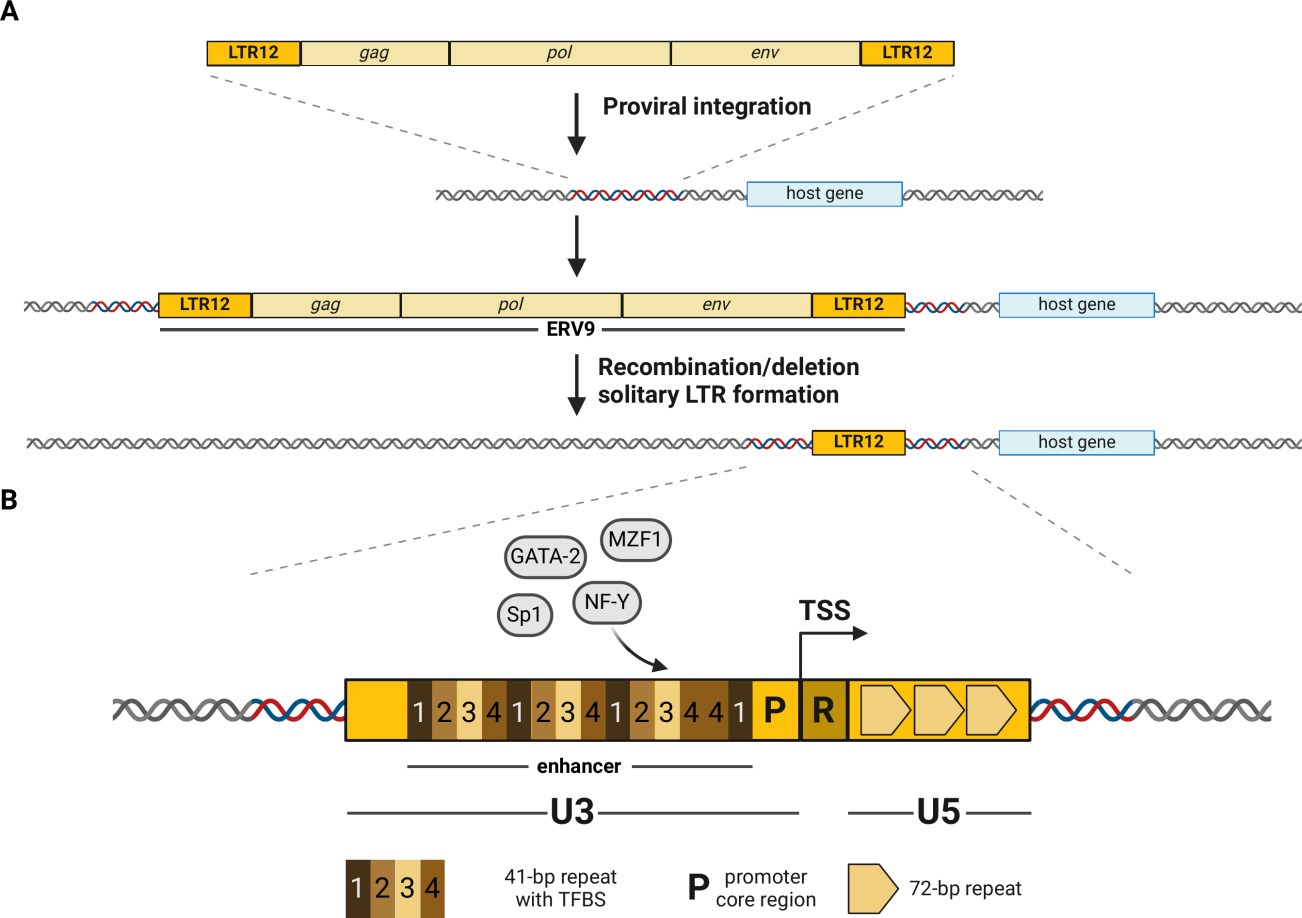
Evolution and sequence characteristics of LTR12 repeats. (**A**) Endogenous retroviruses are the result of retroviral integration events in the host genome that were ultimately fixed in the population. During evolution, the retroviral *gag*, *pol*, and *env* genes are frequently lost due to recombination events of the retroviral long terminal repeats (LTRs). In this case, only so-called solo-LTRs remain. Some solo-LTRs, including several LTR12 repeats, have retained their regulatory activity and may regulate the expression of host genes. (**B**) Arrangement of a prototypical LTR12 repeat in U3, R, and U5 regions, where the U3-R border marks the transcription start site (TSS). The U3 enhancer region is characterized by 5–17 tandem repeats. Each repeat has a length of about 41 nt and can be grouped into one of four closely related subtypes (1, 2, 3, or 4). These repeats comprise recurrent binding sites for transcription factors (TFBS), such as NF-Y, GATA-2, and MZF1. Like U3, the U5 region also varies in length due to varying numbers of 72 bp repeats.

Human endogenous retroviruses (HERVs) have been given many unflattering names: “junk DNA,” “genomic parasites,” “dark matter of the genome,” “parasitic DNA,” etc. This might in part be due to their evolutionary origins. HERVs are descendents of infectious retroviruses that most likely caused disease in our ancestors millions of years ago. One key step of the retroviral replication cycle is the integration of the reverse transcribed viral genome into the genome of the host cell (Fig. 1A). Occasionally, such an integration event may occur in a germ cell. The germ cell may have been directly infected or fused with an already infected cell, e.g., a hematopoietic cell. If the germline integration event is not detrimental to the developing organism, the retroviral genome may be inherited as a novel allele and ultimately be fixed—as an endogenous retrovirus—in the host population (1).

Based on their ancestors and phylogeny, HERVs are subgrouped into Class I (gammaretrovirus-like), Class II (betaretrovirus-like), and Class III (spumavirus-like) elements (2, 3). Over time, HERVs have accumulated numerous mutations and lost their ability to code for infectious viral particles (1). In many cases, they have also lost their typical genome structure, where retroviral genes (*gag*, *pol*, *env*) are flanked by long terminal repeats (LTRs), resulting in only parts of the sequence remaining. In fact, more than 90% of all HERVs are so-called solitary-LTRs (solo-LTRs) where the protein-coding regions have been lost due to homologous recombination events between the LTRs (Fig. 1A) (4). These solo-LTRs have become a significant focus of research due to their potential regulatory role. In exogenous retroviruses, LTRs contain enhancer elements, act as promoters, and harbor transcription start and termination signals, enabling transcription of viral genes. Many endogenous solo-LTRs have retained their enhancer and/or promoter activity and have been co-opted by the host organism to regulate cellular gene expression (4). Closely related solo-LTRs may form regulatory networks that are activated in response to specific stimuli and induce concerted transcriptional responses (5). Notably, the expansion of specific solo-LTR loci that act as cis-regulatory elements can also facilitate adaptive processes and ultimately drive the evolution of new host species (6). Thus, many HERVs, in particular solo-LTRs, are not merely “junk DNA,” but have been co-opted by the host to fulfill important physiological functions.

One fascinating group of solo-LTRs are LTR12 repeats, which comprise a few thousand copies in the human genome. These LTRs are derived from the ERV9 group of endogenous retroviruses, belonging to Class I (2). They have been further subdivided into LTR12, LTR12_v, LTR12B, LTR12C, LTR12D, LTR12E, and LTR12F elements based on specific sequence differences, such as unique insertions and deletions (7, 8). Different LTR12 sublineages and loci may have evolved from independent germline infections by closely related viruses or from a single successful retrovirus that was integrated into the germline multiple times.

In recent years, it has become clear that different members of the LTR12 family share many similarities in their regulation, structure, and function. For example, they are activated in response to viral infection (9–11) or histone deacetylase inhibition (12–18). Moreover, many of them regulate the expression of genes involved in immunity, senescence, and hematopoiesis (Table 1). Thus, the study of LTR12 solo-LTRs provides a valuable opportunity to understand the interplay between ancient retroviral insertions and host genome regulation. In this mini-review, we will summarize our current knowledge of the mechanisms governing LTR12 activity and their downstream effects on cellular gene expression and ultimately on the whole organism.

**TABLE 1:** Functions of selected LTR12 loci

Sub- lineage	Hg38 position	Function/comments	References
LTR12	chr4:99353538–99354277	(Alternative) promoter for *ADH1C*	(16, 19)
LTR12	chr6:32579365–32579821 and chr6:32580133–32580200	May have shaped the evolution of HLA-DR haplotypes	(20–23)
LTR12C	chr1:89127019–89128609	(Alternative) promoter of *GBP2*	(11)
LTR12C	chr1:89272453–89273890	Promoter of *GBP5*	(11, 24, 25)
LTR12C	chr2:180691205–180692425	Promoter of lncRNA *SchLAP1*	(26)
LTR12C	chr2:27110685–27112017	(Alternative) promoter of *CGREF1*	(16, 25)
LTR12C	chr3:115791927–115792364 and/or chr3:115791380–115791922	Promoter of regulatory lncRNA *TST1*	(27)
LTR12C	chr3:85504025–85505239	(Alternative) promoter of short *CADM2* variant	(28)
LTR12C	chr3:189595943–189597160	(Alternative) promoter of *TP63*	(16, 25, 29–31)
LTR12C	chr3:32939501–32941155	(Alternative) promoter of *CCR4*	(25, 32)
LTR12C	chr3:114237141–114238856	Promoter of *ZNF80*	(33)
LTR12C	chr4:76042532–76043850 and/or chr4:76049792–76051220	Potential alternative promoter of *CXCL11*	(32)
LTR12C	chr5:114432500–114433929	(Alternative) promoter of *KCNN2*	(25, 32)
LTR12C	chr5:12573944–12575033	Promoter of *LINC01194/CT49*	(16, 25)
LTR12C	chr5:150845282–150846933	Promoter of *IRGM*	(25, 32, 34)
LTR12C	chr7:12555202–12556721	(Alternative) promoter of *SCIN*	(35)
LTR12C	chr7:84880388–84881736	Major regulator of *SEMA3A*	(28)
LTR12C	chr8:23069937–23071352	Regulator of *TNFRSF10B*	(16, 25)
LTR12C	chr8:83402758–83404051	Promoter of lncRNA LINC01419/PRLH1	(36)
LTR12C	chr9:89479488–89480983	Promoter of *SEMA4D*	(11, 16, 24)
LTR12C	chr9:21959076–21960297 and chr9:21960298–21960419	Partially encoding *CDKN2A-AS1* antisense RNA	(16, 25)
LTR12C	chr11:5293260–5294954	Enhancer upstream of the *HBB* (beta-globin) gene	(37–42)
LTR12C	chr15:99007374–99008797	Promoter of *PGPEP1L*	(16)
LTR12C	chr15:78245272–78246702	(Alternative) promoter of *ACSBG1*	(25, 43)
LTR12C	chr16:68526565–68528236	(Alternative) promoter of *ZFP90*	(35)
LTR12C	chr16:341083–342390	Enhancer in the *AXIN1* gene	(37)
LTR12C	chr20:57348214–57349645	(Alternative) promoter of *RAE1*	(44)
LTR12C	chr22:39357358–39358791	Potential alternative promoter of *SYNGR1*	(43)
LTR12C	chrX:85971257–85972921	Alternative splice acceptor site for *CHM*	(45)
LTR12C	chrX:125255463–125256877	Potential alternative promoter of *TENM1*	(25, 32)
LTR12D	chr14:23635627–23636652	(Alternative) promoter of *DHRS2*	(16, 46)
LTR12F	chr1:155637452–155637661	IFN-γ-responsive enhancer of HERV-K102	(47)
LTR12F	chr6_GL000251v2_alt:3934615–3935104	May have shaped the evolution of HLA-DR haplotypes	(20–23)

## ORIGIN AND EVOLUTION OF LTR12 REPEATS

ERV9/LTR12 is a rather young family of endogenous retroviruses. It invaded the genomes of our ancestors about 30 million years ago (7, 48, 49) before the split of new world and old world monkeys. A major expansion of ERV9/LTR12 loci and the formation of different subfamilies occurred after the divergence of lesser apes from great apes until the divergence of gorillas (7). In agreement with this, many human LTR12C repeats are conserved in the respective syntenic loci of chimpanzees and gorillas, but absent from rhesus macaques and other old world monkeys (49). The proliferation and fixation of LTR12 elements continued until about 6 million years ago, when it ceased for unknown reasons (48). Eventually, the expansion of ERV9 resulted in 4,000–6,000 LTR12 elements in the human genome (26, 50) (http://www.repeatmasker.org), making LTR12 one of the most successful ERV repeats in humans.

## SEQUENCE CHARACTERISTICS OF LTR12 REPEATS

LTR12 repeats vary in length, ranging from about 500 bp (LTR12F) to more than 1,300 bp (LTR12E) (8). These length variations are the result of insertions and/or deletions and contributed to the definition of different LTR12 subsets. For example, LTR12 and LTR12_ differ by an indel of >50 bp, while LTR12D is a variant of LTR12C with several indels (8). Like other retroviral LTRs, the prototypical LTR12 repeat is segmented into U3, R, and U5 regions (Fig. 1B). The U3 and U5 regions are variable in length due to different numbers of tandem repeats termed E elements (ca. 41 bp) and B elements (72 bp), respectively (51). Many LTR12C, LTR12D, and LTR12E repeats harbor an additional extension of about 600 nt at their 5′ end that may have facilitated their exaptation as cis-regulatory elements (49). The R region of LTR12 repeats is relatively short (52–54). Although R sequences of endogenous retroviral LTRs can harbor sequence motifs that act as alternative poly-adenylation signal for cellular transcripts, we are not aware of any LTR12 repeat exerting this function. The border of R with U3 defines the transcription start site (TSS). In transcriptionally active LTR12C loci, the TSS is frequently found within a GCCAGCAGTGGC motif, located at a relative position of 70% downstream of the 5′ end (11, 54, 55). This sequence comprises an inverted repeat (GCCANNNNTGGC), which was shown to be essential for proper start site utilization (56). Furthermore, the sequence surrounding the TSS includes motifs that almost match the consensus sequence BBCABW of an initiator element (*Inr*), where B = C/G/T and W = A/T (57). *Inr* elements can initiate transcription without a functional TATA box (58), and experiments by La Mantia and colleagues suggested that the ERV9 promoter is indeed TATA-less or harbors only a weak TATA box (59). However, many LTR12 repeats contain an AATAAAA motif about 30 nucleotides upstream of the TSS (11) that is required for efficient transcription initiation and has been proposed to act as TATA box (37, 56), at least in some ERV9 LTRs. In line with these findings, truncation experiments identified a region spanning from −70 to +6 relative to the TSS as a minimal promoter region (59).

Apart from this minimal promoter sequence, transcription efficiency is determined by the presence of multiple transcription factor binding sites (TFBS) in LTR12 repeats. For example, a binding site for specificity protein 1 (Sp1) was identified directly upstream of the TATA box-like sequence (59). While only a single ERV9 promoter was analyzed in this publication (59), more recent studies identified TFBS that are conserved in several LTR12 repeats. For example, Ito and colleagues performed an elegant systematic analysis of publicly available ChiP-seq data sets to identify binding sites of 97 transcription factors in HERVs (5). TFBS that are conserved in a substantial fraction of specific endogenous retroviruses were termed HSREs (“HERV/LTR-shared regulatory elements”) by the authors. Interestingly, LTR12, LTR12_, LTR12B, LTR12C, and LTR12D repeats showed comparably high proportions (56%–87%) of HSREs suggesting functional conservation of TFBS in these solo-LTRs.

Many of these TFBS are found within the tandem repeats in the U3 enhancer region that are repeated 5 to 17 times and have been subdivided into four closely related sequences (1, 2, 3, and 4) (37, 38, 59, 60). One important binding site that was experimentally validated in several independent studies is a binding site for nuclear transcription factor Y (NF-Y) (5, 16, 19, 39, 44, 49, 60, 61). This ubiquitously expressed transcription factor is a trimeric complex of NF-YA, B, and C and binds to CCAAT motifs via its NF-YA subunit (62). It maintains regions upstream of TSS in a nucleosome-depleted state (63) and acts as a pioneer factor that promotes chromatin accessibility for other transcription factors (64). CCAAT motifs are highly conserved in the tandem repeats within the U3 enhancer regions of all subfamilies of LTR12 (49). These repeats also contain binding sites for the GATA binding protein (GATA) family of transcription factors (TCTGTATCTAGCT) and G-rich motifs (GTGGGGA) that resemble the binding site for myeloid zinc finger 1 (MZF1) (60). In agreement with this, Yu and colleagues found that NF-Y bound to the CCAAT motif of LTR12 recruits GATA-2 and MZF1 but not GATA-1 or Sp1 (60). Thus, many active LTR12 repeats have the potential to recruit an NF-Y/MZF1/GATA-2 enhancer complex regulating cellular transcription. Since MZF1 and GATA-2 both play key roles in the regulation of hematopoiesis (65, 66), it is tempting to speculate that LTR12 repeats contribute to this process.

In summary, many LTR12 repeats have retained functional motifs, such as TSS or TFBS, that enable them to regulate cellular gene expression.

## CO-OPTED LTR12 REPEATS

### LTR12-derived promoters

**Fig 2:**
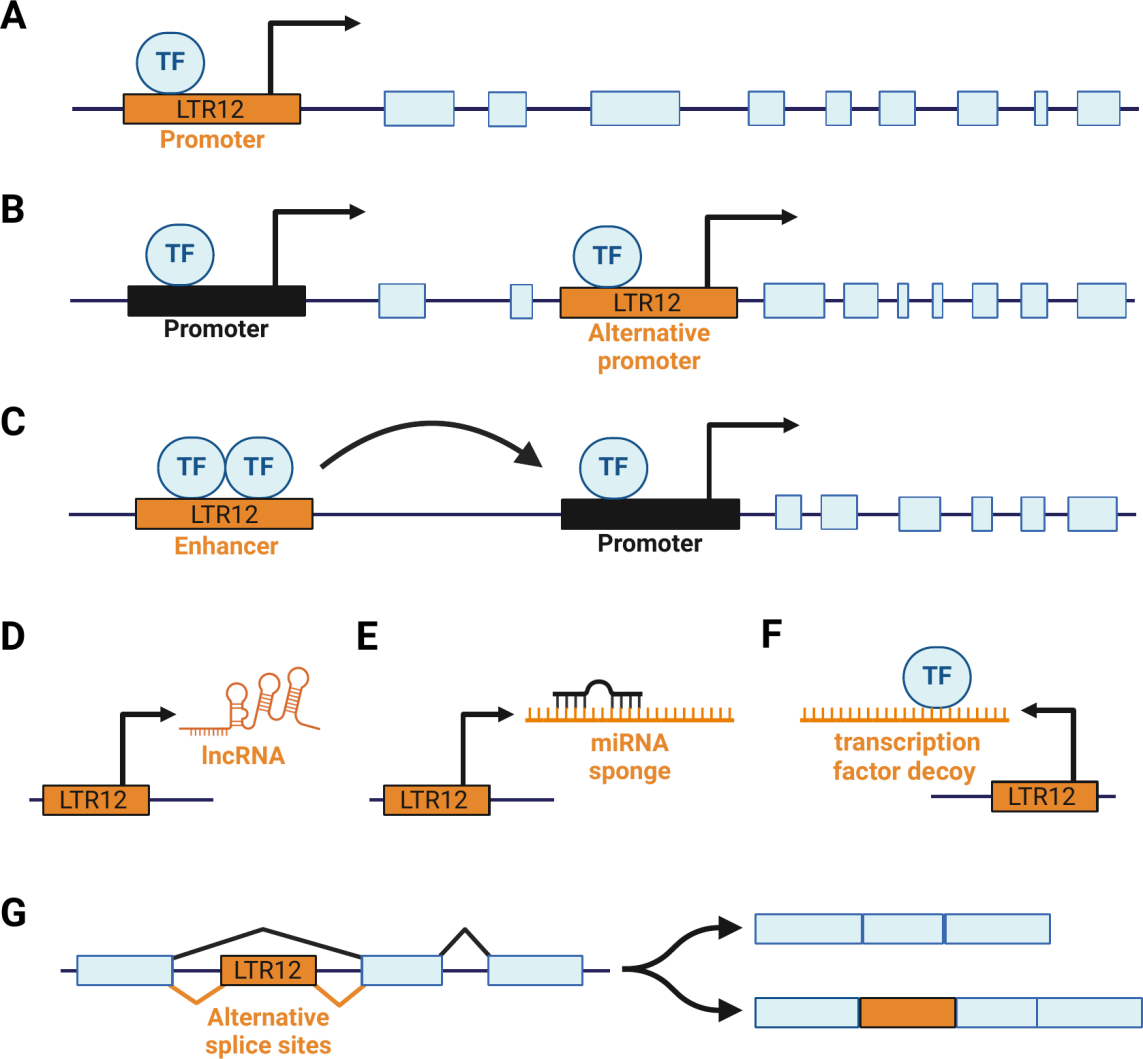
Mechanisms of LTR12-mediated regulation of cellular gene expression. LTR12 repeats can act as (**A**) primary promoters, (**B**) alternative promoters, or (**C**) enhancers for cellular genes. Furthermore, some LTR12 loci code for regulatory RNAs, including (**D**) lncRNAs, (**E**) miRNA sponges, and (**F**) transcription factor decoys. Finally, splice donor or acceptor sites within LTR12 repeats could enable (**G**) alternative splicing of cellular genes.

Given the presence of conserved transcription factor binding sites and transcription start sites, it is not surprising that several LTR12 solo-LTRs have retained their cis-regulatory activity and act as promoters driving cellular gene expression (Table 1) (Fig. 2A). In many cases, retroviral solo-LTRs act as alternative promoters rather than replacing the function of the native primary promoter (Fig. 2B) (24). Still, these alternative promoters enable the host to fine-tune cellular gene expression in certain tissues, during specific developmental stages, or in response to external stimuli. Moreover, alternative promoters can result in the generation of novel transcript variants, further increasing the adaptive repertoire of the cell. For example, LTR12-derived alternative promoters enable the expression of additional mRNA variants and/or protein isoforms of cellular factors, such as DHRS2, GBP2, or CADM2 (11, 16, 28, 46) (Table 1).

A study mapping transcription start sites revealed that treatment with DNA methyltransferase inhibitors (DNMTi) and histone deacetylase inhibitors (HDACi) activates multiple cryptic TSS in LTR12 promoters, primarily of the LTR12C subtype (12). Intriguingly, LTR12C-driven transcriptional regulation was primarily unidirectional in this experimental setup (12). However, as observed for other endogenous retroviral LTRs (67–69), certain LTR12 repeats also promote bidirectional transcription (28, 70). Until today, multiple target genes of LTR12 promoters have been described in human cells. In this section, we will describe a few pairs of LTR12 promoters and their targets in more detail, but refer to Table 1 for a more comprehensive overview.

Two examples of alternative LTR12C-derived promoters are found upstream of *ZFP90* and *SCIN*, encoding for a transcription factor regulating hematopoietic stem cell differentiation (71) and a regulator of cortical actin networks (72), respectively. Brind’Amour and colleagues showed that these alternative promoters are active in human oocytes, where the canonical main promoter is hypermethylated (35). As expected, the primary promoter is used in other cell types, as well as in rat and mouse oocytes, which lack ERV9/LTR12 insertions.

Another interesting example of an LTR12-regulated gene is the immunity-related GTPase family M protein (*IRGM*). This gene codes for an interferon-inducible GTPase that is also known as interferon-inducible protein 1 (IFI1) and participates in the immune response against intracellular bacteria (34, 73). For example, IRGM promotes the elimination of intracellular bacteria by inducing autophagy (73). In the common ancestor of simians, the *IRGM* gene has been pseudogenized due to the insertion of an Alu element that disrupted the open reading frame (34). In the ancestor of Great apes, however, the gene was resurrected by initiating translation at a new start codon directly downstream of the inserted Alu repeat. This reacquisition of the open reading frame coincided with an ERV9 fixation event upstream of the Alu locus. As a result, an LTR12 repeat serves as the functional promoter of this gene in humans (34).

Notably, the expression of two additional immunity-related GTPases, *GBP2* and *GBP5*, is also regulated by LTR12C elements. In humans, these two guanylate-binding proteins (GBPs) are strongly inducible by interferon-gamma (IFN-γ) (11, 74, 75) and restrict a variety of viruses, bacteria, and intracellular parasites (76). For example, GBP2 and GBP5 reduce the infectivity of newly produced human immunodeficiency virus (HIV) particles by interfering with the trafficking and maturation of the viral envelope (Env) protein (11, 77, 78). LTR12C-driven *GBP2/5* expression is induced upon HIV-1-infection (11), suggesting that these two LTR12C repeats contribute to the restriction of HIV-1. Intriguingly, rhesus macaques lack a *GBP5* gene, and their *GBP2* gene that naturally lacks the LTR12C promoter is not responsive to IFN-γ stimulation (11). Thus, it is tempting to speculate that the fixation of LTR12C solo-LTRs conferred a selection advantage to the host since they rendered *GBP2* and *GBP5* IFN-γ-responsive (11).

Infection with HIV also triggers activation of the LTR12C and LTR12D promoters of *SEMA4D* and *DHRS2*, respectively (11). While SEMA4D/CD100 activates T cells, B cells, and dendritic cells (79, 80), DHRS2 regulates p53 signaling (81). In the case of *DHRS2* (also known as *HEP27*), the LTR12D repeat serves as an alternative promoter that drives its transcription in hepatocytes (46). Together, the promoters of *IRGM*, *GBP2, GBP5*, and *SEMA4D* provide striking examples of how the human genome has exapted virus-derived regulatory elements to regulate immune responses against currently circulating pathogens.

### LTR12-derived enhancers

LTR12 elements are also an important source of enhancers (Table 1) (Fig. 2C). In an elegant study, Karrttunen and colleagues performed STARR-seq (self-transcribing active regulatory region sequencing) to identify enhancer elements in colon and liver cancer cell lines that are derived from ERVs or other transposable elements (TEs) (82). In this unbiased approach, fragments of the genome of the cells of interest are cloned into reporter vectors, downstream of a minimal promoter. Consequently, fragments with enhancer activity will boost their own transcription, which can be quantified by paired-end sequencing (83). The authors found that LTR12 and MER11 repeats act as tissue-specific enhancers and were enriched in liver (HepG2) and colon (GP5d) cancer cells, respectively. ChIP-seq in combination with enrichment analyses for transcription factor binding motifs revealed binding of NF-YA to LTR12C-derived enhancers in HepG2 cells (82). Since several of the identified LTR12C repeats were also transcriptionally active, the authors speculate that they may simultaneously act as promoters and enhancers (82, 84).

A few LTR12-derived enhancers have been characterized in more detail (Table 1). One interesting example is an LTR12F element upstream of HERV-K102, a full-length HERV encoding for an Env protein (85). The LTR12F repeat is truncated, lacks its own TSS, and fails to drive efficient expression of a luciferase reporter gene on its own. However, it enhances the activity of the adjacent HERV-K102 5′-LTR in response to IFN-γ stimulation (47). In agreement with these findings, IFN-γ treatment increased chromatin accessibility, as well as H3K27 acetylation, an active enhancer mark (86), across the LTR12F repeat (47). Furthermore, ChIP-seq demonstrated binding of STAT1 and IRF1 to LTR12F upstream of HERV-K102 (47). These two transcription factors are activated in response to immune activation, e.g., upon IFN-γ stimulation (87). IRF1 most likely binds to LTR12F via a motif containing the core binding site AANNGAAA. In contrast, no consensus binding site for STAT1 is present within the LTR12F repeat, and the authors speculate that STAT1 is recruited indirectly, possibly via IRF1 (47, 88). Together, these findings may help explain why HERV-K102 is upregulated under pro-inflammatory conditions, such as HIV-1 infection or systemic lupus erythematosus (85, 89, 90).

Another well-characterized example is an ERV9 LTR at the 5′ boundary of the control region of the human globin gene locus. Transcriptional analyses of the beta-globin locus and LTR reporter assays revealed that the LTR12C enhancer is more active in erythroid (progenitor) cells than in non-erythroid cells (38, 91). In this particular ERV9 LTR, the U3 region comprises 14 tandem repeats of ~40 bp (38, 60). These repeats harbor CCAAT and GATA motifs that are bound by NF-Y and GATA-2, respectively, in erythroid progenitor cells (91). Transcription is initiated within the LTR and has been suggested to enhance far downstream globin gene expression by opening up the chromatin structure (38). Indeed, mutation of the CCAAT motif abrogated NF-Y binding and closed the chromatin structure of the downstream epsilon-gene promoter (60). In addition to a role in opening up the chromatin structure, the ERV9 LTR can recruit and transport transcription factors to the downstream globin genes (38). As described in more detail in the following section, this delivery of transcription factors may involve the binding of ERV9-derived regulatory lncRNAs (92).

### LTR12-derived RNAs with regulatory activity

Apart from acting as classical promoter and/or enhancer elements, LTR12 repeats can modulate cellular gene expression by encoding regulatory RNAs, including long non-coding RNAs (lncRNAs) (Fig. 2D through F). For example, Hu et al. showed that the interaction of LTR12 enhancers with NF-YA, GATA-1, and RNA polymerase II (pol II) is strengthened by LTR12-derived lncRNAs (92). In a so-called tracking and transcription mechanism (93), the resulting complex composed of LTR12-DNA, NF-YA, GATA-1, pol II, and LTR12-RNA migrates along the DNA until it ultimately loops with its target gene, enhancing its expression *in cis* (92). While the authors focus on the LTR12 enhancer complex regulating globin gene expression, they hypothesize that many additional LTR12-derived transcripts may modulate long-range LTR enhancer activity (92).

Another example of an LTR12-derived regulatory transcript was identified in cancer cells. Hepatocellular carcinoma cells express an lncRNA termed tumor-specific transcript 1 (TST1) that originates from an LTR12C promoter and is associated with poor survival rates (27). Silencing of TST1 reduced the proliferation of hepatoma cell lines, while its overexpression enhanced tumor growth in mice. TST1 most likely accelerates cell proliferation by sequestration of the microRNA miR‐500a‐3p (Fig. 2E) (27). In line with this model, miR‐500a‐3p inhibits hepatoma cell proliferation (27). Although miR‐500a‐3p overexpression and TST1 depletion result in an overlapping pattern of differentially expressed genes (27), the exact factors that are regulated by miR‐500a‐3p and/or TST1 and determine cell proliferation remain to be determined.

TST1 is not the only LTR12-derived RNA modulating cell proliferation. Xu et al. identified transcripts that originate from the U3 region of LTR12 repeats that regulate cell cycle progression (94). These transcripts cover the tandem repeats of U3, including the CCAAT binding sites for NF-Y. They most likely originate from LTR12 elements found in introns of cellular genes, such as *AXIN1* or *HLA-DRB* (94), and are co-transcribed via read-through transcription. Since intronic LTR12 repeats have been inserted in forward and reverse orientation (95), it is not surprising that both sense and antisense LTR12 U3 transcripts have been detected (94). Intriguingly, the antisense (but not sense) transcripts are able to bind the transcription factors NF-Y, p53, and Sp1 (94) (Fig. 2F). Interactions between transcription factors and RNA have also been described for non-retroviral transcripts before (96, 97). In fact, a recent study suggests that more than half of all transcription factors bind RNA, most likely via a domain that is similar to the arginine-rich motif in HIV-1 Tat (98). Whether a similar domain is also involved in the binding of transcription factors to LTR12 transcripts remains to be determined. Notably, the binding of LTR12-derived RNA seems to be relatively specific since antisense RNAs derived from the ERV-K111 LTR failed to sequester NF-Y. The authors show that the LTR12-mediated sequestration of NF-Y reduces NF-Y-dependent transcription of cyclins B1 and B2, thereby inhibiting cell cycle progression (94). The sequestration of p53 by LTR12 antisense transcripts did not rescue cell cycle progression, although p53 is an important negative regulator of the cell cycle that is known to suppress cyclin B expression (99). In line with these findings, non-malignant cells express relatively high levels of LTR12 U3 antisense transcripts, while highly proliferative cancer cells express less (94).

Notably, p53 is not only sequestered by LTR12-derived transcripts, but also represses the expression of at least one LTR12C repeat (36). This LTR12C locus acts as promoter and partially encodes for a lncRNA that has been termed PRLH1 (p53-regulated lncRNA for homologous recombination repair 1). Due to its negative regulation by p53, this lncRNA is highly expressed in cancer cells with inactivating p53 mutations. Like other prototypical LTR12 elements, PRLH1-LTR12 harbors multiple CCAAT repeats that render it responsive to NF-Y (36). As its name implies, PRLH1 regulates DNA repair. Mechanistic analyses revealed that it binds to ring finger protein 169 (RNF169) via two GCUUCA motifs. RNF169 is an E3 ubiquitin ligase and DNA repair protein that promotes homologous recombination while inhibiting nonhomologous end joining (100). The binding of PRLH1 to RNF169 increases its stability, further promoting homologous recombination (36). Thus, PRLH1 is an example of an LTR12-derived lncRNA that regulates the repair of dsDNA breaks and promotes the proliferation of p53-mutated cancer cells (36).

### LTR12-derived splice sites

Since retroviral genomes typically harbor multiple splice donor and acceptor sites, their fixation in human genes may also be associated with changes in the cellular splice pattern. However, ERV-mediated splicing events are often deleterious and selected against, partly explaining why the members of most ERV families are more frequently found in intergenic regions rather than introns (95). Furthermore, usage of intragenic ERV splice sites may be suppressed if ERVs are inserted in the antisense orientation, possibly as a result of antisense/sense mRNA annealing (95). In line with this, ERV9 repeats are not known to be involved in any mRNA splicing events when oriented in the antisense direction (95). In contrast, van de Langemaat et al. identified 12 splice events within ERV9 elements in the sense orientation (95). Since LTRs do not usually contain any splice sites, it is not surprising that these twelve ERV9-derived splice sites are all located outside the LTR.

Nevertheless, an LTR12C repeat within the *CHM* gene has been shown to harbor a splice acceptor site (45). This alternative splice site has most likely been acquired after fixation of the respective LTR and results in a truncated isoform of the CHM protein (isoform b). Since this isoform is produced to higher levels in colon, testis, and lung tumors compared to adjacent healthy tissues, the respective transcript has been proposed as potential tumor marker (45).

Thus, although LTR12-derived splice sites seem to be relatively uncommon in the human genome, they may contribute to alternative transcript variants in healthy and malignant cells.

## REGULATION OF LTR12 ACTIVITY

Given the gene regulatory activities of many LTR12 elements, it is not surprising that LTR12 activity itself is tightly regulated. Like other transposable elements, ERV9/LTR12 activity is suppressed by a variety of mechanisms at the pre- and post-transcriptional level. These include epigenetic changes, such as trimethylation of histone H3 lysine 9 (H3K9me3) or CpG/DNA methylation. Since ERV suppression has already been summarized in detail in other excellent review articles (101–103), we here focus on inhibitory pathways that may be specific to ERV9/LTR12 and/or were specifically characterized in the context of this ERV family.

LTR12C repeats show G-to-A mutation patterns indicative of restriction and editing by apolipoprotein B mRNA editing enzyme catalytic subunit 3 (APOBEC3) proteins (104). More specifically, LTR12C elements are characterized by an enrichment of GG-to-AG mutations on the positive strand that is consistent with the editing pattern of APOBEC3G (104). Unexpectedly, LTR12C and LTR12D loci show a higher rate of G-to-A mutations on their negative strand, which cannot be readily explained by APOBEC3-induced mutations (105). Nevertheless, these *in silico* analyses show that the evolution and nucleotide sequences of LTR12C elements and/or their exogenous ancestors were most likely influenced by APOBEC3 proteins in the past.

**Fig 3:**
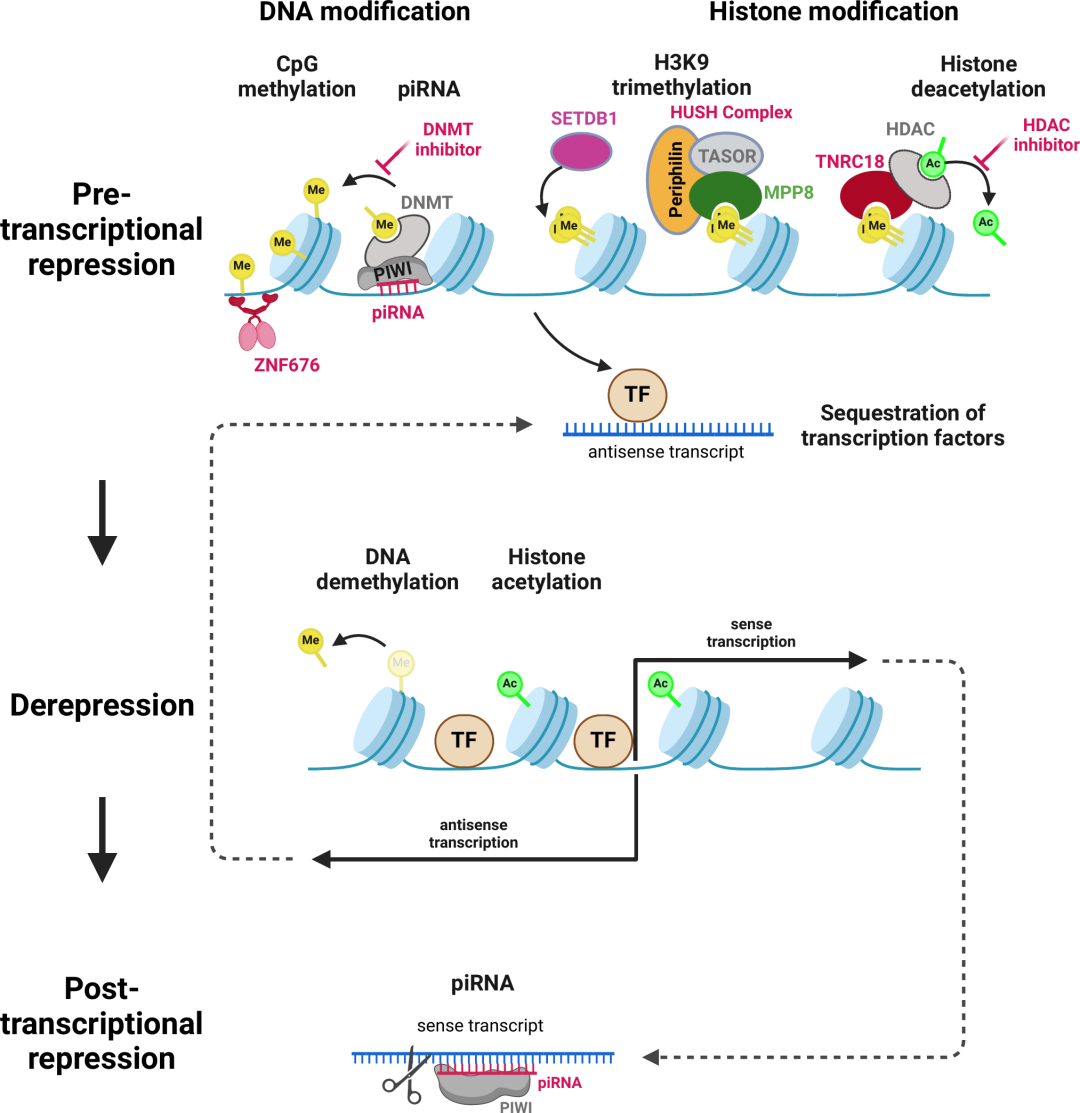
Mechanisms of LTR12 regulation. At the pre-transcriptional level (top), LTR12 repeats are repressed by DNA marks (e.g., CpG methylation) and histone marks (e.g., H3K9 trimethylation). DNA methylation is associated with binding of the KRAB zinc finger protein ZNF676 and potentially piRNA/piwi complexes. The HUSH complex, including MPP8, TASOR, and periphilin contributes to LTR12 restriction by recruiting the histone methyltransferase SETDB1 for H3K9 trimethylation and stabilizing repressive chromatin. The adaptor protein TNRC18 is also involved in LTR12 restriction, recruiting HDACs to H3K9 marks. Finally, TE-derived antisense transcripts may suppress LTR12 transcription via a sequestration of transcription factors, such as NF-Y. At the post-transcriptional level (bottom), piRNA/piwi complexes may potentially induce the targeted degradation of LTR12-derived transcripts.

One factor contributing to APOBEC3 resistance is the GC content of a nucleotide sequence (106). Intriguingly, LTR12C repeats and other LTR12 elements are more CpG-rich than most other ERV LTRs (26), and all ERV9/LTR12 subfamilies show a high level of CpG methylation (49, 61) (Fig. 3). This suppressive methylation is maintained in primordial germ cells (49), although these epigenetic marks are usually erased and reset during this stage of development (107, 108). During early embryonic development, DNA methylation of LTR12 repeats, particularly in the 5′ extension of LTR12C, is closely associated with ZNF676 (49). This KRAB zinc finger protein restricts the expression of a subset of chimeric LTR12 gene transcripts (49). Notably, ZNF676 is humanoid-specific, suggesting that both LTR12 and ZNF676 appeared at approximately the same time and co-evolved with each other (49). However, Iouranova and colleagues hypothesized that ZNF676 did not restrict the expansion of LTR12 repeats in the human genome, but rather facilitated it, due to a tight regulation during specific developmental stages or in specific tissues (49).

In line with a key role of DNA methylation in LTR12 restriction, treatment with DNA methyltransferase inhibitors (DNMTi) induces the activation of numerous cryptic start sites in LTR12 elements (12, 14). A very similar set of TSS is activated by histone deacetylase inhibitors (HDACi), such as SAHA/vorinostat or SB939 (12–18), demonstrating that histone deacetylation contributes to their repression. However, combined treatment with both DNMTi and HDACi is required for full activation. Notably, H3K9me3 and histone deacetylation of LTR12 repeats are mechanistically linked by the chromatin regulator trinucleotide-repeat-containing 18 (TNRC18), which senses H3K9me3 and recruits HDAC/Sin3/NCoR complexes (43). Depletion of TNRC18 results in an activation of cryptic TSS within LTR12 repeats. In agreement with targeting and restricting LTR12 solo-LTRs, TNRC18-bound sites are enriched for CCAAT motifs (43), i.e., the repetitive NF-Y binding sites found in the U3 region of LTR12 (49). Apart from TNRC18, the H3K9me3 reader M-phase phosphoprotein 8 (MPP8) was also shown to be involved in ERV9 restriction, as siRNA-mediated depletion of MPP8 and its interaction partner TASOR increased LTR12 expression (109, 110). Together with periphilin, TNRC18 and MPP8, are part of the human silencing hub (HUSH) complex, which promotes the deposition of additional repressive H3K9me3 marks via the histone N-methyltransferase SET domain bifurcated 1 (SETDB1) (111).

Apart from the epigenetic regulators described above, piwi-interacting RNAs (piRNAs) have also been implicated in the restriction of LTR12C repeats. These small non-coding RNAs are primarily found in germ cells, where they form complexes with so-called piwi-subfamily argonaute proteins and guide them to complementary TE-derived RNAs, inducing their degradation. In addition to this post-transcriptional inhibition, piRNA/piwi complexes also bind to their target sequences in the nucleus, where they induce DNA methylation (112). In case of LTR12C repeats, piRNA-mediated inhibition has been demonstrated for pre- and postnatal stages of spermatogenesis (113).

Finally, LTR12 transcripts may also restrict their own synthesis via a negative feedback mechanism. As described above, LTR12-derived antisense transcripts bind transcription factors, such as NF-Y and Sp1 (94) (Fig. 2F). Since these factors also drive LTR12-mediated gene expression, their sequestration may reduce the activity of LTR12 promoters.

Together, the above-mentioned examples illustrate that LTR12 activity is tightly regulated and involves a few inhibitory mechanisms (e.g., ZNF676) that are largely specific to this group of transposable elements. Despite the multilayered suppression, however, ERV9/LTR12C transcripts are detectable in a variety of healthy tissues (114), and deregulated under pathophysiological conditions such as infections and cancer.

## CONSEQUENCES OF LTR12 ACTIVATION IN INFECTION AND CANCER

Aberrant LTR12 activity during infections and cancer may be a side effect of disease or directly contribute to pathogenesis. In this section, we describe a few LTR12-regulated pathways and mechanisms that may (co)determine the outcome of viral infections and cancer, respectively.

### Infection

**Fig 4:**
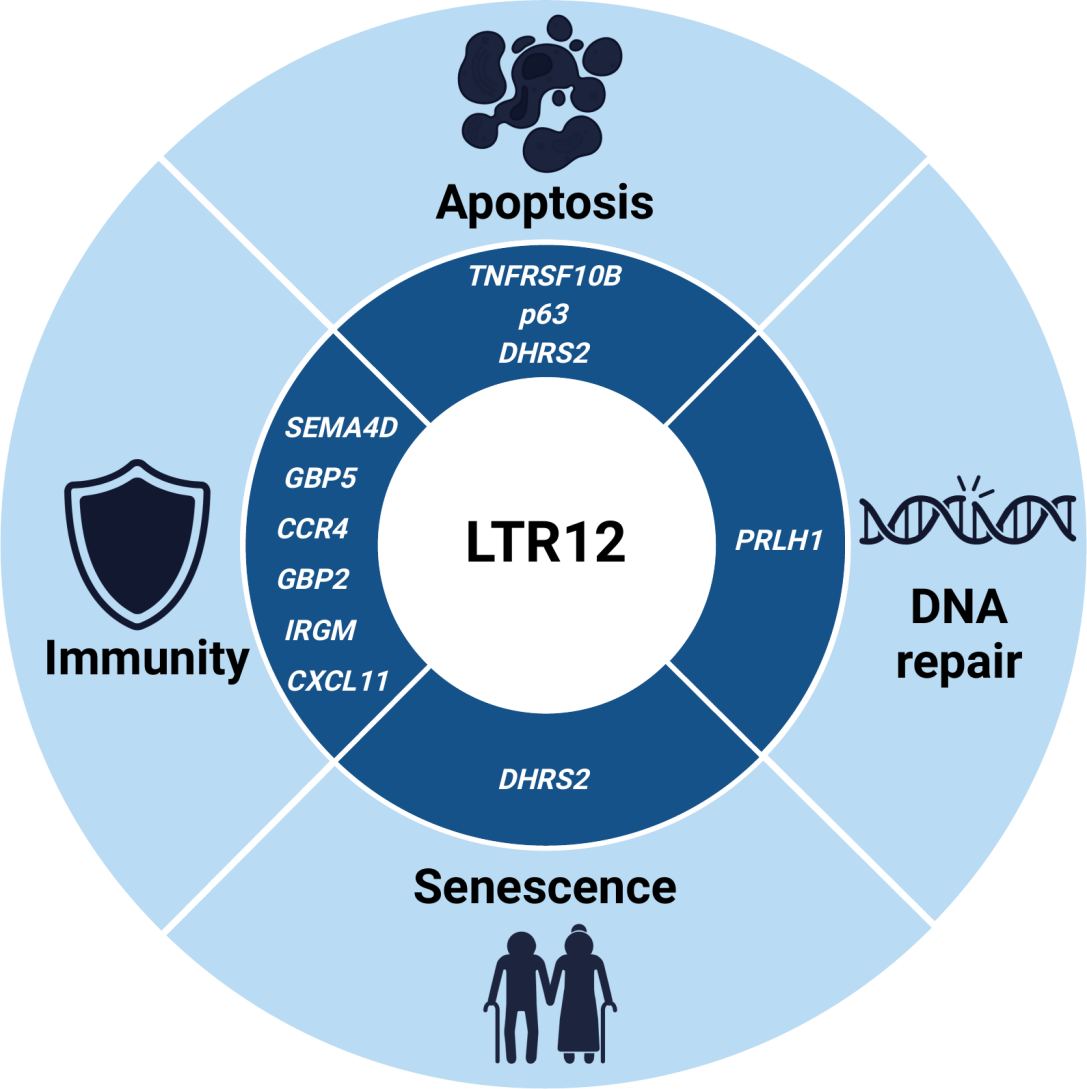
LTR12-regulated genes protect us from infections and cancer. Several LTR12 target genes are involved in the immune response to infection (left). These include genes encoding cytokines and their receptors (CXCL11 and CCR4), immune cell activators (SEMA4D), and components of the cell-intrinsic immune response against intracellular pathogens (IRGM, GBP2, and GBP5). Moreover, LTR12 repeats may exert anti-tumor activities by regulating proteins and RNAs involved in DNA repair (PRLH1), apoptosis (TNFRSF10B, p63, and DHRS2), and/or senescence (DHRS2).

Two major lines of evidence suggest that LTR12 elements play a key role in regulating immune responses: First, numerous ERV9/LTR12 loci are activated in response to infections with various pathogens, including HIV (10, 11, 115) and human cytomegalovirus (HCMV) (9). In fact, ERV9/LTR12 is the most enriched ERV family among HIV-induced transposable elements (10, 11). Second, several immunity-related genes are regulated by LTR12-derived enhancers or promoters. These include modulators of immune cell activation (e.g., *SEMA4D*), chemokines, and their receptors (e.g., *CXCL11*, *CCR4*), as well as large IFN-inducible GTPases with broad activity against viruses, bacteria, and/or intracellular parasites (e.g., *GBP5*) (Table 1) (Fig. 4). Intriguingly, LTR12-mediated transcription is also triggered in uninfected bystander cells (11) and may protect them from infection by inducing an antiviral state. Furthermore, this observation indicates that LTR12 repeats are activated in response to a soluble factor, e.g., a cytokine. Apart from a direct regulation of immunity genes, it is tempting to speculate that LTR12-derived transcripts are also sensed by pattern recognition receptors, further boosting the induction of an antiviral state, as previously observed for other transposable elements (116). In line with this, previous studies in human and avian systems revealed that RIG-I and TLR3 can sense ERV-derived dsRNA that may form when sense and antisense transcripts hybridize (117, 118). However, at least in leiomyosarcoma cells, the induction of LTR12 transcription via HDAC inhibition was not sufficient to trigger an interferon response (13). Still, the presence of multiple LTR12-derived promoters and enhancers regulating immunity genes suggests that LTR12 loci have been co-opted as a regulatory network that controls part of the transcriptional response to infection.

### Cancer

Apart from viral infections, LTR12 activity is also deregulated in cancer cells (16, 55, 82). Here, LTR12 repeats may act as a double-edged sword. On the one hand, LTR12-derived or LTR12-regulated factors may promote tumor growth. For example, an LTR12C locus acts as promoter for the lncRNA SWI/SNF complex antagonist associated with prostate cancer 1 (*SchLAP1*), which is overexpressed in about 25% of prostate cancers, where it suppresses the anti-tumor activity of the chromatin-modifying SWI/SNF complex (26, 119, 120). *SchLAP1* depletion and overexpression experiments revealed that this lncRNA is essential for tumor invasiveness and metastasis i*n vivo* (119). Similarly, transcription of multiple LTR12C loci is increased in hepatocellular carcinoma and associated with higher risk of tumor recurrence (55). One LTR12C-derived transcript that is highly expressed in p53-mutated hepatocellular carcinoma and promotes tumor cell proliferation is the lncRNA PRLH1 described above (36, 121). Of note, aberrant LTR12C activity was mostly observed in HBV-positive hepatocellular carcinomas (55), suggesting that viral infection may at least in part contribute to LTR12C induction, as also observed for other viral pathogens (9–11, 115).

On the other hand, some LTR12-regulated transcripts may protect us from tumor expansion. For example, an LTR12D promoter drives the expression of *DHRS2* (11, 46), which stabilizes the tumor-suppressor protein p53 (81). p53 stabilization can lead to apoptosis and induce a transient cell cycle arrest, enabling DNA repair or result in cellular senescence, i.e., a permanent cell cycle arrest (122). All three mechanisms prevent uncontrolled proliferation of tumor cells. A protective cell cycle arrest may not only be induced by the LTR12D–DHRS2–p53 pathway, but also by antisense RNAs derived from U3 regions of LTR12 elements. Xu and colleagues propose that these antisense transcripts sequester transcription factors, such as NF-Y or Sp1, that are essential for cellular proliferation (94). In male germ cells, an LTR12C repeat also induces the expression of a specific isoform of p63, another member of the p53 family (29). In line with a protective pro-apoptotic role *in vivo*, p63 expression is frequently lost in testicular cancer (29). In agreement with a contribution to tumor formation, an unbiased analysis of somatic single nucleotide variations (SNVs) in cancer patients revealed a significant enrichment of mutations in ERV9/LTR12 repeats (123). Together, these examples illustrate that individual LTR12 loci regulate central pathways in tumorigenesis and are deregulated in different tumor entities.

## UNANSWERED QUESTIONS

While a lot of progress has been made in the characterization of individual LTR12 loci and their regulatory activity, several open questions remain. For example, the exact pathways governing the derepression of multiple LTR12 repeats under pathophysiological conditions, such as viral infection, have remained unclear. Why are LTR12 elements activated in both infected cells and uninfected bystander cells? Is LTR12 activity a common signature of many infections that is largely independent of the pathogen? Have some viruses evolved mechanisms to limit LTR12-mediated immune activation? Furthermore, the global downstream consequences of LTR12 activation on different cell types remain to be defined. Does the activation of regulatory LTR12 repeats generally induce an antiproliferative, pro-apoptotic and/or antiviral state? To address this question, a targeted activation of LTR12 repeats via CRISPR activation (CRISPRa) will be useful. In a recent study, Ohtani and colleagues established a CRISPRa/dCas9-SunTag-VP64-based approach that simultaneously trans-activates more than 600 LTR12C repeats (32). This tool will be valuable to investigate whether global LTR12 activation induces transcriptional responses that protect us from cancer and/or infection.

Future studies should also further explore a targeted modulation of LTR12 activity as a potential therapeutic approach, primarily in the context of cancer. For example, Beyer and colleagues have already shown that HDACi-mediated activation of ERV9/LTR12 can prime tumor cells for apoptosis. They demonstrated that the proapoptotic cytokine TRAIL in combination with LTR12C-mediated induction of its receptor TNFRSF10B efficiently kills testicular cancer cells (25). HDACi treatment also promoted apoptosis of cancer cells in combination with cisplatin, suggesting that LTR12-mediated activation of apoptosis may help suppress tumor growth (16, 25). Since HDAC inhibition may not specifically de-repress LTR12 elements, but also other transposable elements, more targeted approaches are desirable. These could include a modulation of LTR12-specific repressors, such as ZNF676 or sequence-specific approaches (e.g., CRISPRa), that activate only a subset of LTR12 repeats. More specifically, nucleotide alignments may identify sequence motifs that are unique to LTR12 repeats with beneficial effects in tumor therapy. Based on these motifs, sgRNAs can be designed that allow CRISPR-mediated activation of beneficial LTR12 loci, while preventing the activation of LTR12 repeats that drive the expression of oncogenes (see previous section). Apart from inducing LTR12 activity, oligonucleotides mimicking LTR12 sequences with tumor-suppressive activity may also be used. For example, Xu and colleagues showed that short U3-derived oligodeoxynucleotides can be used as decoys for transcription factors, such as NF-Y, and inhibit the growth of different human tumor cell lines *in vitro* and in xenograft mouse models (94). Finally, tumor-specific LTR12-derived protein isoforms should also be explored as potential targets for diagnosis, prognosis, and/or treatment of cancer, as previously proposed for other HERV families (17).

In summary, LTR12 repeats provide a fascinating example of remnants of once-infectious retroviruses that have been repurposed to establish a network of gene regulatory elements. Future studies will reveal whether and how we can exploit this network further for the diagnosis, prevention, or treatment of disease.
